# Controlling SIRT1 expression by microRNAs in health and metabolic disease

**DOI:** 10.18632/aging.100184

**Published:** 2010-08-05

**Authors:** Jiyoung Lee, Jongsook Kim Kemper

**Affiliations:** Department of Molecular and Integrative Physiology, University of Illinois, Urbana-Champaign, IL 61801, USA

**Keywords:** FXR, SHP, miR-34a, p53, SIRT1, and metabolic syndrome

## Abstract

SIRT1 is a NAD^+-^dependent deacetylase implicated in longevity and diverse physiological processes. SIRT1, as a key mediator of beneficial effects of caloric restriction, regulates lipid and glucose metabolism by deacetylating metabolic regulators, as well as histones, in response to nutritional deprivation. Here we discuss how SIRT1 levels are regulated by microRNAs (miRs) which are emerging as important metabolic regulators; the recently identified nuclear receptor FXR/SHP cascade pathway that controls the expression of miR-34a and its target SIRT1; and a FXR/SIRT1 positive feedback regulatory loop, which is deregulated in metabolic disease states.  The FXR/miR-34a pathway and other miRs controlling SIRT1 may be useful therapeutic targets for age-related diseases, including metabolic disorders.

## Introduction

Disruption in metabolic homeostasis and over accumulation of metabolites, cholesterol, bile acids, triglycerides (fat), or glucose, play causative roles in the development of metabolic disorders, such as, atherosclerosis and related heart disease, fatty liver, obesity, and diabetes. The NAD^+^-dependent SIRT1 deacetylase plays a critical role in maintaining metabolic homeostasis which affects aging so that SIRT1 increases life spans in most organisms, including mammals [1-3]. Despite extensive studies on SIRT1 function and its beneficial metabolic effects, how the expression of SIRT1 is regulated under normal conditions and how SIRT1 levels are decreased in metabolic disease states remain unclear. In this review, we survey recent studies showing how SIRT1 expression is regulated at the post-transcriptional level, focusing on microRNAs (miRs) which have recently emerged as important cellular regulators [4-6]. We also review recent studies showing that the nuclear receptor FXR/SHP cascade pathway which controls expression of miR-34a and its target SIRT1 in normal conditions and is dysregulated in metabolic disease states.

### SIRT1: a key regulator in cellular metabolism

Caloric restriction (CR) was shown to increase life span and promote survival in yeast, worms, flies, rodents and perhaps primates [1,2]. SIRT1 mediates the beneficial metabolic effects of CR in an NAD^+^-dependent manner by deacetylating and altering the activities of transcriptional factors which regulate metabolic genes [1,2,7]. SIRT1 deacetylates and activates transcript-tional ability of metabolic regulators, such as PGC-1α, p53, Foxo 1, NF-κB, LXR, and FXR that are involved in lipid and glucose metabolism, inflammation, mitochondrial biogenesis, and energy balance [1,2,8-12]. In addition, SIRT1 was shown to be recruited to the promoter of metabolic target genes and suppress their transcription [13,14]. It was reported that SIRT1 is associated with the promoter of PPARγ, a key adipogenic factor, and suppresses PPARγ transcription by recruiting the corepressors, NcoR1 and SMRT [14]. SIRT1 was reported to bind to the UCP 2 gene promoter and inhibit its transcription in pancreatic β-cells, resulting in increased ATP production and insulin secretion [13]. SIRT1 was also shown to improve insulin sensitivity by repressing transcription of protein tyrosine phosphatase 1B, a major negative regulator of insulin action, via histone deacetylation [15]. Beneficial metabolic functions of SIRT1 have been demonstrated in studies using small molecule activators and transgenic mice that are null for SIRT1 or overexpress SIRT1 [16-20]. The natural compound resveratrol and the synthetic compound SRT1720 are activators of SIRT1 and have been shown to ameliorate insulin resistance, increase mitochondrial content, improve metabolic profiles, and increase survival in mice fed a high-fat diet [16-18]. Transgenic mice expressing SIRT1 were shown to be resistant to body weight gain and ameliorated insulin resistance and glucose intolerance in these mice compared to wild-type control mice [20]. Further, transgenic mice expressing moderate amounts of SIRT1 were also shown to protect livers from diet-induced metabolic damage [12,21]. Consistent with these reports, in liver-specific SIRT1 null mice challenged with a high fat diet, fatty acid metabolism was altered and the development of fatty livers and inflammatory responses were promoted [19,22]. Loss of function studies also showed that SIRT1 decreases endothelial activation in hypercholesterolemic ApoE-/- mice without affecting endothelium-dependent vasodilatation [23]. All these recent studies demonstrate that SIRT1 is a key regulator of cellular metabolism and mediates beneficial metabolic effects.

### MicroRNAs: emerging metabolic regulators

MicroRNAs (miRNAs) are small (approximately 22 nt) non-coding RNAs that control gene expression [4-6]. MiRs are transcribed from DNA by RNA polymerase II as hairpin precursors which are further processed to mature forms [4-6]. MiRs bind to the 3'-untranslated region (UTR) of target mRNAs and inhibit their expression by causing mRNA cleavage or inhibition of translation. Approximately 30% of all human genes are thought to be regulated by miRs [5,6] and indeed, miRs control gene expression in diverse biological processes including development, differentiation, cell prolifera-tion, and apoptosis. Recent studies have demonstrated crucial roles of miRNAs in the regulation of cellular metabolism [24-32]. MiRs are involved in lipid and glucose metabolism in major metabolic tissues, such as, liver, pancreas, adipose, and muscle as summarized in Table 1. Mir-122 is the most abundant miR in the liver and plays important roles in a wide variety of liver functions ranging from cholesterol metabolism, liver cancer, stress responses, viral infection, to circadian regulation of hepatic genes [24,28,29]. MiR-33 has been shown to contribute to the regulation of cholesterol homeostasis by targeting the cholesterol transporter genes, ABCA1 and ABCG1 [25,26]. Our group recently reported that miR-34a targets hepatic SIRT1 and, interestingly, expression of miR-34a was highly elevated and SIRT1 levels were decreased in fatty livers of diet-induced obese mice [30]. MiR-34a was also shown to suppress insulin secretion in pancreatic β-cells [33]. The roles of miR-375 in pancreatic islet functions, especially in insulin gene transcription, insulin secretion, and islet cell growth, are also well established [31,32]. Mir-27 and miR-378 were reported to control adipocyte differentiation and lipid synthesis, respectively [34,35]. MiR-223 was shown to regulate glucose uptake in cardiomyocytes and miR-696 to regulate mitochondria biogenesis and fatty acid oxidation in gastrocnemius muscle [36,37]. In line with their critical functions, miRs are often underexpressed or overexpressed in disease states [4,6,24,28,30,38-40]. Recent studies have shown that restoring miRs or downregulating miRs using antisense miR inhibitors, called antagomirs, has improved transcriptional and biological outcomes, demonstrating that miRs are promising therapeutic targets [4,24,38].

### Down-regulation of SIRT1 by microRNAs

Consistent with its critical roles in diverse biological processes, the regulation of SIRT1 expression is fine tuned at multiple levels, including transcriptional, post-transcriptional, and post-translational levels. The general regulation of SIRT1 activity and expression has been thoroughly reviewed in excellent articles [1-3,41] and, therefore, this review focuses on the regulation of SIRT1 expression by miRs (Table 2). MiR-34a was first identified as a posttranscriptional regulator of SIRT1 in the regulation of apoptosis under cellular genotoxic stress in human colon cancer HCT116 cells [42]. MiR-34a binds to the 3' UTR of SIRT1 mRNA in a partial complementary manner and represses its translation but does not affect mRNA degradation [30,42]. Our group further reported that miR-34a targets hepatic SIRT1 in the regulation of cellular metabolism in human hepatoma HpeG2 cells and in mouse liver in vivo using adenoviral-mediated overexpression of miR-34a [30]. Remarkably, we observed that miR-34a levels are highly elevated and SIRT1 protein levels are substantially decreased in the fatty livers of both diet-induced obese mice and the leptin-deficient ob/ob mice [30]. These findings are in line with recent studies showing that miR-34a is the most elevated miR in livers exhibiting nonalcoholic steatohepatitis, a spectrum of nonalcoholic fatty liver diseases in humans [39]. Other miRs also target SIRT1. In response to nutritional availability, miR-132 was shown to downregulate SIRT1, resulting in activation of inflammatory pathways in adipose tissues [43]. MiR-199a was identified as a negative regulator of SIRT1 and HIF1a, a key mediator of hypoxia [44]. Low oxygen tension results in acute downregulation of miR-199a in cardiac myocytes and in porcine heart and this reduction is required for upregulation of its targets, HIF-1a and SIRT1 in response to decreased oxygen [44]. Interestingly, a recent study showed that SIRT1 protein levels are much higher in mouse embryonic stem cells (ESCs) than in differentiated tissues and that miRNAs, miR-181a and b, miR-9, miR-204, miR-199b, and miR-135, post-transcriptionally down-regulate SIRT1 during mouse ESC differentiation and maintain low levels of SIRT1 expression in differentiated tissues [45].

**Table 1.** MicroRNAs regulating cellular metabolism in major metabolic tissues.

**MicroRNA**	**Direct targets[putative]**	**Functions in Metabolism (references)**	**Tissues (cultured cells)**
**miR-33**	ABCA1, NPC1	Cholesterol homeostasis (25, 26)	Liver (HepG2)
**miR-34a**	SIRT1	lipid metabolism, promotes fatty liver (30)	
**miR-370**	Cpt1a	Fatty acid and triglyceride biosynthesis (29)	
**miR-122**	CAT-1 ADAM17	Hepatic lipid metabolism (24, 29) Circadian gene expression (28)	
**miR-34a**	VAMP2	B-cell exocytosis (33)	Pancreatic Islets (MIN6, INS-1)
**miR-124a**	Foxa2	Intracellular signaling in pancreatic β-cell (27)	
**miR-375**	MTPN	Regulates catecholamine release Inhibits insulin secretion (31, 32)	
**miR-27a**	[PPARγ, C/EBPα]	Inhibits adipocyte formation, Down-regulated during adipogenic differentiation (34)	(Adipocytes, 3T3-L1, ST2)
miR-378/378*	[Ribosomal proteins]	Upregulates adipocyte differentiation and lipid synthesis (35)	
**miR-223**	Glut4	Glucose uptake and insulin resistance (36)	Muscle Gastrocnemius (Cardiomyocyte, C_2_C_12_)
**miR-696**	[PGC1α]	Muscle metabolism, mitochondria biogenesis and fatty acid oxidation (37)	

**Table 2.** MicroRNAs targeting SIRT12.

**MicroRNA**	**Sequences of microRNAs **	**Size (nt)**	**Biological functions (references)**
**miR-34a**	5'-uggcagugucuuagcugguugu-3'	22	Hepatic lipid metabolism (30) Islet β-cell exocytosis (33) Cell apoptosis (42)
**miR-132**	5'-uaacagucuacagccauggucg-3'	22	Stress-induced chemokine production (43)
**miR-199a**	5'-cccaguguucagacuaccuguuc-3'	25	Hypoxia preconditioning (44)

**Figure 1.**
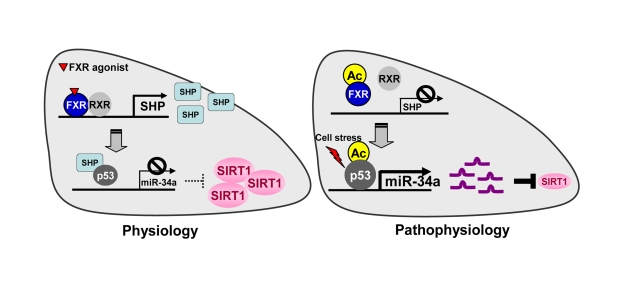
The FXR/SHP pathway controlling miR-34a and SIRT1 expression. Under normal conditions, activation of FXR signaling induces the metabolic repressor SHP in liver.  SHP is then recruited to the miR-34a promoter and inhibits binding of the key activator p53 to the DNA, resulting in decreased miR-34a expression.  Inhibition of miR-34a results in increased hepatic SIRT1 levels. In contrast, under pathophysiological conditions such as fatty livers of obese mice, the dysregulated FXR/SHP pathway due to highly elevated FXR acetylation no longer inhibits transcription of miR-34a.  The dysregulated FXR/SHP pathway, along with acetylation of p53 due to cellular stress under metabolic disease states, result in elevated miR-34a expression, which contributes to decreased SIRT1 levels.

### A novel FXR/SHP/miR-34a pathway controlling SIRT1 levels

The nuclear bile acid receptor, Farnesoid X Receptor (FXR), plays an important role in maintaining lipid and glucose levels by regulating expression of numerous metabolic genes mainly in the liver and intestine [46]. Consistent with its important metabolic functions, disruption of the FXR gene in transgenic mice was associated with metabolic diseases, including hypercholesterolemia, cholesterol gallstone disease, fatty liver, and type 2 diabetes [46-49]. Activation of FXR in diabetic obese mice improved metabolic outcomes by reducing serum glucose and lipid levels [50]. Although both FXR and SIRT1 have been shown to be critical for hepatic metabolism and activation of both proteins improves metabolic outcomes in diet-induced obese mice [17,18,46,47,50], it was unknown whether the expression and activity of these two proteins are coordinately regulated. In recent studies, we found that FXR positively regulates hepatic SIRT1 expression by inhibiting expression of miR-34a [30]. As shown in Figure 1, under normal conditions, miR-34a levels are down-regulated by a nuclear receptor cascade pathway involving FXR and orphan nuclear receptor and metabolic repressor, Small Heterodimer Partner (SHP) [51,52]. Upon induction by activated FXR, SHP is recruited to the miR-34a promo- ter and suppresses its transcription by inhibiting the promoter occupancy of p53, the key activator of the miR-34a gene [53]. Subsequently, inhibition of miR-34a contributes to increased expression of SIRT1. This FXR/SHP pathway was also shown to play a crucial role in the regulation of hepatic bile acid synthesis by inhibiting the rate-limiting bile acid synthetic enzyme CYP7A1 [51,52] and to suppress fatty liver formation by inhibiting the key lipogenic activator SREBP-1c [54]. Our group has identified molecular mechanisms by which SHP inhibits its target genes by coordinately recruiting chromatin modifying repressive cofactors, including HDACs, G9a metyltransferase, and Brm-containing Swi/Snf remodeling complex [55-57]. Consistent with these previous findings, we observed recruitment of HDACs to the miR-34a promoter in mouse liver after treatment with the synthetic FXR agonist, GW4064 (not shown). In contrast, in fatty livers of obese mice, the FXR/SHP pathway is dysregulated such that miR-34a levels are highly elevated, which contributes to reduced SIRT1 levels [30]. Interestingly, activation of FXR signaling in obese mice by daily treatment with GW4064 for 5 days or by hepatic expression of FXR using adenoviral delivery decreased miR-34a levels and restored SIRT1 levels [30]. Consistent with a critical role for FXR in positively controlling SIRT1 through the inhibition of miR-34a, miR-34a levels were indeed elevated and SIRT1 protein levels are substantially decreased in FXR null mice [30]. Our findings suggest an intriguing link among FXR activation, decreased miR-34a levels, increased SIRT1 levels, and beneficial metabolic outcomes.

### A positively interacting FXR/SIRT1 regulatory loop

**Figure 2.**
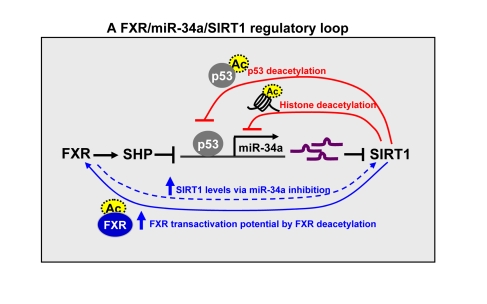
**A FXR/SIRT1 positive-feedback regulatory loop.**  The expression and activity of FXR and SIRT1 are mutually and coordinately regulated.   SIRT1 positively auto-regulates its own expression by inhibiting miR-34a via deacetylation (as indicated by dotted circles) of p53 and histones at the miR-34a promoter (short loop) and by increasing transactivation potential of FXR via deacetylating the FXR (long loop).  SIRT1 also increases FXR expression and activity via deacetylation of PGC-1α. FXR in turn positively regulates hepatic SIRT1 expression by inhibiting miR-34a which targets SIRT1.

In the FXR/SHP/miR-34a pathway, FXR positively regulates hepatic SIRT1 levels by inhibiting transcription of the miR-34a gene. These findings, along with previous studies showing the p53/miR-34a/SIRT1 feedback loop [42,58], suggest intriguing regulatory loops controlling SIRT1 expression (Figure 2). In the short regulatory loop, SIRT1 positively auto-regulates its own expression by deacetylating p53 and histones at the miR-34a promoter, resulting in suppression of miR-34a [9,30,42,53,58]. In the long regulatory loop, SIRT1-mediated deacetylation of FXR increases FXR's transactivation ability by increasing binding of the FXR/RXR heterodimer to DNA resulting in induction of SHP and repression of miR-34a expression [11,30]. We observed that FXR acetylation is dynamically controlled by p300 acetylase and SIRT1 deacetylase under normal conditions, and remarkably, FXR acetylation levels are highly elevated in fatty livers of obese mice [11]. Interestingly, treatment daily with the SIRT1 activator resveratrol for 1 week or adenoviral-mediated hepatic expression of SIRT1 substantially reduced FXR acetylation with beneficial metabolic effects [11]. These results are consistent with the idea that the transactivation activity of FXR is low in obese mice due to highly elevated FXR acetylation, which contributes to increased expression of miR-34a. Subsequently, elevated miR- 34a suppresses expression of SIRT1, which then further decreases FXR activity, resulting in a vicious FXR/miR-34a/SIRT1 regulatory loop in metabolic disease states. In addition to deacetylation of FXR, SIRT1 has been implicated as a positive regulator of the expression and activity of FXR. During fasting, PGC-1α was shown to increase expression of the FXR gene and function as a coactivator of FXR [59]. Since SIRT1 deacetylates and increases PGC-1α activity [8], SIRT1 should increase FXR expression and activity by enhancing PGC-1α activity. All these recent studies strongly suggest that the expression and activity of these two proteins are mutually and coordinately regulated.

### Concluding remarks

Because of SIRT1's anti-aging properties and its beneficial effects on a wide range of age-related disease [1-3,21], it has been intensively studied. SIRT1 levels were reported to be decreased in liver, muscle, and adipose tissues of diet-induced obese mice in vivo as well as in cultured cell models of insulin resistance [15,30,60], but the underlying mechanisms remain unclear. The discovery of the FXR/miR-34a pathway controlling SIRT1 levels provides a partial explanation since elevated miR-34a levels in obese mice contribute to decreased SIRT1 levels [30]. Based on these findings, together with the development of effective inhibitors of miRs, the antagomirs [4,24,38], it will be interesting to see whether the reduction of elevated miR-34a in fatty livers of obesity improves transcriptional profiles of metabolic genes and metabolic outcomes. Also, it will be important to understand how the FXR/SIRT1 regulatory network is dysregulated in metabolic disease states which likely involves altered cellular kinase signaling pathways that post-transcriptionally affect SIRT1 and FXR levels and activities. Development of drugs that target the FXR/miR-34a pathway and other miRs controlling SIRT1 expression may lead to novel therapeutic options for treating age-related metabolic disease including fatty liver, obesity and type II diabetes.
